# The effects of exclusive versus non-exclusive breastfeeding on specific infant morbidities in Conakry

**Published:** 2009-04-03

**Authors:** Fatoumata Binta Diallo, Linda Bell, Jean-Marie Moutquin, Marie-Pierre Garant

**Affiliations:** 1 Département de Santé Publique, Université de Montréal, Montréal, Québec, Canada; 2 Département de Sciences cliniques, Université de Sherbrooke, Sherbrooke, Québec, Canada; 3 Centre de Recherche Clinique étienne-Le Bel du CHUS, Sherbrooke, Québec, Canada

## Abstract

**Background:**

This study examines the effect of exclusive versus non-exclusive breastfeeding on specific infant morbidities from birth to nine months, in Conakry (Guinea).

**Method:**

A cross-sectional study was conducted on 1,167 mother-infant pairs who visited one of 20 immunization centres in Conakry for vaccination between the 45^th^ and 270^th^ days of the child’s life. Two data sources were used: the infant health book and an orally administered questionnaire completed with the mother. Data analyses included univariate cross-tabulations and multivariate logistic regression models to estimate the effect of breastfeeding on infant morbidity.

**Results:**

Exclusive breastfeeding decreased with the infant’s age. At six months of age, the proportion of infants who were exclusively breastfed was only 15.5%. After adjusting for the infant’s age, and the interaction between the type of breastfeeding and the infant’s age, exclusive breastfeeding significantly protected the infants against many of the studied morbidities (OR: 0.28, CI: 0.15–0.51) and specifically against diarrhoea (OR: 0.38; 95% CI: 0.17 – 0.86), respiratory infections (OR: 0.27; 95% CI: 0.14 – 0.50), and low growth rate (OR: 0.11; 95% CI: 0.02 – 0.46), but not for otitis, urinary infection, or meningitis.

**Conclusion:**

This investigation confirmed the protective effects of exclusive breastfeeding on some specific infant’s morbidities during the first nine months of life. The results of this study are of great importance for the development of an information program designed to encourage the exclusive breastfeeding among the mothers of Conakry, Guinea.

## Background

Infant feeding methods are a major determinant of infant nutritional status, which, in turn, affects infant morbidity and mortality. Among feeding methods, breastfeeding is of particular importance because this practice is fundamental for survival, growth, development, health, and nutrition of infants [1]. Each year, 5.6 million infants die because they do not receive adequate nutrition [2]. The world’s health authorities recommend exclusive breastfeeding of all infants until six months of age [3-5]. In spite of all the efforts deployed either as information, education, or training campaigns to promote mother’s milk as the best food for the infant, the prevalence of exclusive breastfeeding remains low [6-9]. Non-exclusive breastfeeding is an important cause of infant morbidities [10–13]. This association has especially been observed in developing countries, but has also been noted in the developed world [14]. Multiple studies have evaluated the effects of breast milk on infants’ health [10, 11, 13, 15] but such studies have rarely been conducted in Africa where infant-feeding practices are more diversified [16, 17]. To our knowledge, no study examining the relationship between infant feeding practices and infant morbidities has been undertaken in Guinea.

In an investigation carried out in Conakry (Guinea), it appeared that breastfeeding was highly practiced (nearly 88.5%) for a long period of time but exclusive breastfeeding was less widespread [6]. Breastfeeding practices are more diversified and are characterized by the late initiation of breastfeeding, the administration of substances other than maternal milk, and the introduction of weaning foods within one month following the infant’s birth. These various practices of infant feeding have an important impact in terms of public health, particularly infant morbidity. In Guinea, 44% of infants under five years of age receive oral rehydration because of diarrhoea and 15% have respiratory infections [18]. We think it is important to investigate the relationship between these breastfeeding practices and infant morbidities in the Guinean context. The aim of our study was to examine the effects of exclusive versus non-exclusive breastfeeding on specific infant morbidities from birth to nine months, in Conakry, Guinea. Specific morbidities investigated included diarrhoea, respiratory infections, otitis, urinary infection, meningitis, and low growth. In addition, we examined the role played by other variables in the association between morbid conditions and the type of breastfeeding.

## Method

This was a cross sectional study of all mother-infant pairs, with infants who were less than nine months of age, who attended any one of 20 immunization centres in Conakry, Guinea, between October and December 2005. All mothers who agreed to participate were included. However, women whose infants were HIV-positive were excluded. Women were considered the mother of the child if they were the biological mother or if they had been caring for the child since his/her birth.

We used a non-probabilistic sampling. Our sample size calculations were based on the Clement et al. (1999) study in which 198 infants were followed from birth to six months [11]. In this study, the incidence of diarrhoea in exclusively breastfed infants amounted to 6.5 and to 12.6 in children who did not receive maternal milk. Furthermore, we assumed a 5% type I error probability and an 80% power. Using these numbers, we obtained a sample size of 231 infants that would be necessary to detect the effect of breastfeeding on the probability of morbidities.

Three kinds of variables were studied. The dependent variable was the presence of at least a morbidity affecting the infant for any period of time before enrolment in the study. Selected morbidities were diarrhoea, respiratory infections, otitis, urinary infection, meningitis, and low growth. The independent variable was the type of breastfeeding from birth to the time of enrolment in the study. This was categorized as exclusive breastfeeding (mother’s milk only, with the exclusion of all other food or drink) and non-exclusive breastfeeding. We studied other variables to check their potential effect on infant morbidity. These included the infant’s age and gender, and whether or not the infant was born preterm, together with the mother’s age, parity, marital status, education, occupation, and household income and size.

Two data sources were used: the infant health book and an orally administered questionnaire to the mother. The infant health book is a medical document that pregnant women receive at their first antenatal consultation. The health book is divided into two sections: antenatal consultation and child monitoring and it contains medical antenatal information and post natal details on the infant health status up to three years of age.

In Conakry, the vaccination follow-up of an infant is done at health centres and at the Institut de Nutrition et de Santé de l’Enfant on the 45^th^ day and on the 3^rd^, 6^th^, and 9^th^ month. Immunization centres where chosen for the recruitment of participants as they provided access to the greatest number of mother-infant pairs. Before data collection, eligible mothers were given explanations concerning the study and, upon verbal consent to participate, they were asked to provide their infant’s health book and answer additional questions. Interviews and data collection were carried out by 20 health professionals (one per center) who had a university-level education and had been trained by the study staff about the study. Data gathering activities were supervised by the principal investigator.

The data was carried out using a questionnaire (Appendix A) that contained closed- and open-ended questions. The first part of the questionnaire was completed using the infant health book. This permitted the collection of all information concerning feeding history, infant morbidity, infant’s growth curve, and socio-demographic characteristics (infant’s age and gender, mother’s age, parity, and gestational age). The second part pertained to the mother characteristics such as marital status, education, mother’s occupation, household income, and household size. For reliability testing, the questionnaire was pretested with on a random selection of 25 subjects from five centres in Conakry.

All questionnaires that were correctly administered and fulfilled the inclusion criteria were included in the analysis. We did a validation of the data acquisition on 10% of the questionnaires — that is 116 questionnaires without finding incomplete or invalid data. Data analyses included simple univariate cross-tabulations as well as multivariate logistic regression models to estimate the effects of type of breastfeeding on infant morbidity. The data was initially presented in frequency distributions (proportions, means, and standard deviations). We used the Student T test for continuous variables and the Chi-square or Fisher test for categorical variables to check relationship between infant morbidity and socio-demographical variables. We used odds ratio to calculate the association between types of breastfeeding and morbidity with 95% confidence interval. To control for potential confounding factors, multivariate models were also tested and associated with infant morbidity in the previous analyses. An alpha of less than 0.05 (P<0.05) was considered statistically significant. All analyses were done using SPSS system software (version 14.0).

This research was approved by the ethics committees of the Health Ministry of Guinea and Sherbrooke’s research centre (‘Comité d’éthique de la recherche en santé chez l’humain du Centre hospitalier universitaire de Sherbrooke’). All immunization centres that took part in this study gave their approval. The participating mothers gave their verbal assent. None of the questionnaire was accessible to any other person than the investigator and all the respondents were considered anonymous.

## Results

Table 1 describes the study population. The sample consisted of 1,167 mother-infant pairs. The mean age of the infants was 145±75 days (about 4^1/2^ + 2 months). The proportion of boys and girls was equivalent (583 and 584, respectively). Among recruited infants, 40 (3.4%) were born prematurely. The averaged age of mothers was 25.6± 6.0 years and 35.6% (418) were primiparas. The majority were married (975; 83.5%). Almost half of them did not have any formal education (544; 46.6%) and 454 (38.6%) were unemployed. Slightly more than three-quarters of participants had a monthly household income higher than 210,000 fg (918; 78.7%), the equivalent of $57 (cdn). The household size was on the average 6.7±3.5 persons.

**Table 1:** Socio-demographic characteristics of infants and their mothers in the sample

Variables			n=1167(%)	Mean (SD)
**Infants**				
	**Age (days)**			145.7 ± 75.9
	**Gender**			
		Male	583 (50.0)	
		Female	584 (50.0)	
	**Gestational age < 37 weeks**		40 (3.4)	
**Mothers**				
	**Age (years)**			25.6 ± 6.0
	**Parity**			
		Primipara	418 (35.8)	
		Multipara	749 (64.2)	
	**Marital status**			
		Married	975 (83.5)	
		Single	178 (15.3)	
		Divorced	8 (0.7)	
		Widowed	6 (0.5)	
	**Education**			
		Illiterate	544 (46.6)	
		Primary	214 (18.3)	
		Secondary	281 (24.1)	
		Vocational school	90 (7.7)	
		University	38 (3.3)	
	**Occupation**			
		Unemployed	454 (38.9)	
		Sales	229 (19.6)	
		Dressmaker	144 (12.3)	
		Hairdresser	136 (11.7)	
		Office worker	63 (5.4)	
		Other^1^	141 (12.1)	
	**Household income^2^**			
		£ 200,000 fg	249 (21.3)	
		> 200,000 fg	918 (78.7)	
	**Household size**		
		6.7 ± 3.5

1 : Artist, Artisan, Gardener, Carpenter, Knitter, Gas pump attendant, Student, waitress.

2 : $1 C = 3500 Guinea franc (fg).

In our sample, we found that 523 (44.8%) infants were exclusively breastfed at any period before their enrolment in the study while 644 (55.2%) infants were not. Only 61 (15.5%) infants were exclusively breastfed up to six months of age (180 days). Among infantile morbidities, respiratory infections were the most frequently encountered (39.8%), followed by diarrhoea (22.6%), otitis (17.9%), low growth (5.6%), urinary infection (0.6%), and meningitis (0.2%) (Table 2).

**Table 2:** Frequency of morbidity among infants from birth to the day of enrolment into the study

**Morbidity (n =1167)**				**n (%)**
	No-morbidity		482 (41.3)
	At least one morbidity		685 (58.7)
**Specific morbidity (n=685)^$^**				
	Diarrhoea		264 (22.6)
	Respiratory infections		464 (39.8)
	Otitis		209 (17.9)
	Urinary infection		7 (0.6)
	Low growth		65 (5.6)

$ Artist, Artisan, Gardener, Carpenter, Knitter, Gas pump attendant, Student, waitress.

Table 3 shows the distribution of infants with at least one morbidity according to socio-demographic variables. The only variable which was found significantly associated with morbidity was infant age. There was no significant association with any other characteristics (i.e., infant’s gender, mother’s age, parity, gestational age, marital status, mother’s education, mother’s occupation, household income, and household size).

**Table 3:** Frequency of morbidity among infants from birth to the day of enrolment into the study

Variables			Morbidity (n = 685)	P-value
Infant age (mean ± SD)			167 ± 74.87	0.001^*^
**Gender**					
	Male n (%)	352 (60.4)	0.244
	Female n (%)	333 (57.0)	
Gestational age < 37 weeks n (%)			22 (55.0)	0.629
Mother’s age (mean ± SD)			25.69 ± 6.26	0.561
Mother primipara n (%)			249 (59.6)	0.651
**Marital status**				**n (%)**s	
	Married		566 (58.1)	
	Single		109 (61.2)	
	Divorced		6 (75.0)	0.642
	Widowed		4 (66.7)	
**Mother’s education**				n (%)	
	Illiterate		309 (56.8)	
	Primary		133 (62.1)	
	Secondary		168 (59.8)	0.691
	Vocational school		54 (60.0)	
	University		21 (55.3)	
**Mother’s occupation**				n (%)	
	Unemployed		259 (57.0)	
	Sales		135 (59.0)	
	Dressmaker		82 (56.9)	0.396
	Hairdresser		91 (66.9)	
	Office worker		39 (61.9)	
	Others		79 (56.0)	
**Household income**				n (%)	
	≤ 200000 fg		137 (55.0)	0.18
	> 200000 fg		548 (59.7)	
Household size (mean ± SD)			6.89 ± 3.53	0.09

* P≤0.05

Table 4 shows adjusted associations between type of breastfeeding and morbidity. Infants who were exclusively breastfed had a significantly decreased risk of contracting at least one morbidity compared to infants who were non-exclusively breastfed (OR: 0.28; 95% CI : 0.15 – 0.51). These results were adjusted for infant age and the interaction between type of breastfeeding and infant’s age. When specific morbidities were studied, we found that exclusive breastfeeding seems to protect infants against diarrhoea (OR: 0.38; 95% CI: 0.17 – 0.86), respiratory infections (OR: 0.27; 95% CI: 0.14 – 0.50), and low growth rate (OR: 0.11; 95% CI : 0.02 – 0.46), compared with non-exclusive breastfeeding. However, such difference was not observed for otitis, urinary infection, and meningitis.

**Table 4:** Prevalence of infant morbidity by type of breastfeeding. (exclusive breastfeeding vs. non-exclusive breastfeeding)

At least one morbidity	Diarrhoea	Diarrhoea	Respiratory infections	Low growth
Exclusive breastfeeding n (%)	233 (44.6)	63 (12.0)	150 (28.7)	13 (2.5)
Non exclusive breastfeeding n (%)	452 (70.2)	201 (31.2)	314 (48.8)	52 (8.1)
Adjusted odds ratio^*^ (95%CI)	0.28 (0.15-0.51)	0.38 (0.17-0.86)	0.27 (0.14-0.50)	0.11 (0.02-0.46)

* Adjusted for infant’s age, and interaction between type of breastfeeding and infant’s age

## Disscussion

In our study we found that exclusive breastfeeding conferred protection against infantile morbidity at least up to 270 days (nine months). Previous studies have also observed such protective effect of exclusive breastfeeding on morbidity [10, 11, 13, 19, 20]. In looking at the relationship between the type of breastfeeding and specific morbidities, we found a decreased risk of diarrhoea and respiratory infections in infants who were exclusively breastfed as already reported by others [10, 11, 15, 21-23]

However, contrary to reports from other studies [12, 24], the data presented does not support the influence of type of breastfeeding on the frequency of otitis in this population. One possible explanation is that these studies were carried out in developed countries. Other reasons for this discrepancy could be that we did not differentiate between degrees of breastfeeding (i.e., we did not evaluate according to the quantity of mother’s milk consumed by the infant) and the retrospective nature of our data. The small proportion of infants with urinary infections and meningitis in this study did not allow us to examine the effects of type of breastfeeding on these illnesses.

Our findings indicate that the prevalence of exclusive breastfeeding until six months of age was 15.5%. This rate is consistent with another sample of the Guinea population [6, 7]. However, the prevalence of exclusive breastfeeding in our study is below that found in most developing countries, where the average rate is 39% [7]. Variations in study methods and the definition of breastfeeding used in each of these studies may explain this difference.

Our findings must be viewed in line of the limitations of our study. Because the breastfeeding data were collected retrospectively, true effects may have been obscured. Furthermore, relationships between types of breastfeeding were probably underestimated because some infants died due to fatal morbidities, and were, therefore, not included in our sample (as their mothers did not visit the immunization centres to have their infants vaccinated). We did not take into account the difference between the infants who were raised by their biological mothers and those raised by others in the data analysis and this could involve some biases in infants’ morbidities. In addition, the non-probabilistic sampling methods used for this study also involved selection bias.

A noteworthy aspect of this study was its methodology. By employing a pretested questionnaire and training interviewers, we controlled for some of the biases. We also gathered data on potential confounders and applied multivariate modelling to adjust for them. In addition, the large sample size and the fact that it is the first study in Guinea to evaluate the relationships between type of breastfeeding and infant health strengthen the study.

## Conclusion

According to our results, the risk of morbidity is reduced by close to 70% when a child is exclusively breastfed. Exclusive breastfeeding protected against serious morbidities (diarrhoea, respiratory infections, and low growth) in the first six months of life even after adjusting for confounding variables. However, the type of breastfeeding had no effect on otitis, urinary infection, and meningitis in this study. In Conakry, few mothers exclusively breastfeed during the first six months of an infant’s life. It is therefore necessary to promote interventions aimed at exclusive breastfeeding. The Heath Ministry in Guinea will be one of the main users of these investigation results. We hope this will lead to the development of public information programs on how breastfeeding practices influence infant health.

Two useful implications emerge from this study. First, we suggest the improvement of the information strategies, education, and training concerning the advantages and disadvantages associated with the multiples types of breastfeeding in Guinea. The second implication suggests that exploratory study on the association between types of breastfeeding and infants’ morbidities may be useful in future research. This will help by seriously considering morbidities that involve infants’ death and also investigation into association between the types of breastfeeding and others morbidities, which we could not show as a result of the retrospective data used in our study.

## Competing interests

The authors declared that they have no conflicts of interest.

## Authors’ contributions

**FBD:** is the principal author and also directed the data gathering and writing of the paper.

**LB** and **JMM:** took part more in the conception of methodology and interpretation of results. They also contributed in the coherence of the text and language used.

**MPG:** supervised the statistical analysis and the interpretation of result.

## Acknowledgements

This study was supported in part by the Canadian International Development Agency and Centre de Recherche Clinique étienne-Le Bel du CHUS. We thank the Guinean health authorities for their support. We also thank the children and mothers who participated in this study for their cooperation and patience.
